# 4-Hydroxyisoleucine Alleviates Macrophage-Related Chronic Inflammation and Metabolic Syndrome in Mice Fed a High-Fat Diet

**DOI:** 10.3389/fphar.2020.606514

**Published:** 2021-01-21

**Authors:** Jiali Yang, Yunhui Ran, Yonghui Yang, Shuyi Song, Yahong Wu, Yuanming Qi, Yanfeng Gao, Guodong Li

**Affiliations:** ^1^ School of Pharmaceutical Sciences, Zhengzhou University, Zhengzhou, China; ^2^ School of Life Sciences, Zhengzhou University, Zhengzhou, China; ^3^ School of Pharmaceutical Sciences (Shenzhen), Sun Yat-sen University, Guangzhou, China

**Keywords:** 4-hydroxyisoleucine, insulin resistance, obesity, macrophages, inflammation

## Abstract

In obesity, macrophages and other immune cells accumulate in organs affected by insulin, leading to chronic inflammation and insulin resistance. 4-Hydroxyisoleucine (4-HIL) is a non-protein amino acid found in fenugreek seeds. 4-HIL enhances insulin sensitivity, but its mechanism is still unclear. In this study, 4-HIL intervention reduced weight gain, liver steatosis, and dyslipidemia; moreover, it increased systemic insulin sensitivity and improved insulin resistance in mice. Importantly, after administration, the accumulation of M1 like CD11c^+^ macrophages and inflammation in the liver and adipose tissue were reduced in the mice. 4-HIL also reduced the proportion of CD11c^+^ macrophages among bone marrow-derived macrophages, which were induced *in vitro*. These observations demonstrate a new role of 4-HIL in insulin resistance in hepatocytes and adipocytes. 4-HIL inhibits obesity-related insulin resistance by reducing inflammation and regulating the state of M1/M2 macrophages.

## Introduction

Insulin resistance is a key part of the etiology of type 2 diabetes, and obesity is clearly the most common cause of insulin resistance in humans (Moller and Kaufman, 2005). As a result of the ongoing global obesity epidemic, the prevalence of related metabolic diseases has increased (Jastreboff et al., 2019). One of the hallmarks of obesity in humans and rodents is chronic inflammation of adipose tissue, and liver and skeletal muscle (Haiyan et al., 2003; Weisberg et al., 2003; Nicolas et al., 2010; Pillon et al., 2013). In this obesity-induced tissue inflammatory response, the accumulation of proinflammatory macrophages significantly increases, especially in adipose tissue and the liver (Pingping et al., 2010; Lackey and Olefsky, 2016).

Studies have shown that anti-inflammatory treatment and the disruption of important genes in proinflammatory response can improve insulin sensitivity in obese animals (Kim et al., 2001; Yuan et al., 2001). The dominant immune cell type causing inflammation in obese and T2DM islets is the macrophage (Ying et al., 2020). Macrophage recruitment and polarization are key to obesity-induced inflammation and insulin resistance. Many previous studies have shown that macrophage accumulation is increased in adipose tissue, liver tissue, and muscle tissue in obese individuals, particularly with a large increase in pro-inflammatory M-1-like Cd11c^+^ macrophages (Lumeng et al., 2007; Olefsky and Glass, 2010; Bijnen et al., 2018). Removing CD11c^+^ cells *in vivo* can improve obesity-related insulin resistance, suggesting that chronic tissue inflammation plays an important role in obesity-related insulin resistance (Patsouris et al., 2008). Studies have shown that the accumulation of adipose tissue macrophages is positively correlated with the degree of obesity in mice and humans (Weisberg et al., 2003). However, treatment options for immune cells aimed at preventing the development of insulin resistance and type 2 diabetes remain limited. There is an urgent need for new and promising treatments for obesity-related metabolic diseases in order to control and possibly reverse the progression of the disease.

Chinese traditional medicine has a long history in Asian countries (Stone, 2008). 4-HIL is a special amino acid that does not exist in mammalian tissues, but only in some plants, especially fenugreek (Fowden et al., 1973; Sauvaire et al., 1984). Fenugreek, a leguminous plant, has been used as part of traditional medicine for the treatment of diabetes, and 4-HIL has been proven to be one of the active components of fenugreek (Sharma, 1986; Broca et al., 2000). Fenugreek has anti-hyperglycemia and anti-dyslipidemia effects in a diabetic animal model (Narender et al., 2006; Singh et al., 2010; Rawat et al., 2014). Sauvaire et al. studied the structure of 4-HIL (Broca et al., 2000). The authors described that the molecule with three chiral centers existed in fenugreek seeds in the form of two dienantiomers. The main non-enantiomers with the 2S, 3R, and 4S accounted for approximately 90% of the total 4-HIL in seeds, followed by 2R, 3R, and 4S. The main 4-HIL isomers (2S, 3R, 4S) extracted from fenugreek seeds were the most effective insulin sensitizers among the 12 structure-related amino acids tested (Broca et al., 2000). In addition, 4-hydroxyisoleucine potentiates insulin secretion in a glucose-dependent manner. (Broca et al., 1999), although the mechanism needs to be further studied. In this paper, 4-HIL is a drug with the (2S, 3R, 4S) configuration obtained by microbial enzyme transformation. The purity of the product was 98.3%, the stability was good, and the oral bioavailability was high. In this study, we evaluated the therapeutic effect of 4-HIL on obesity and metabolic disorders, and explored the potential mechanism of this effect.

## Materials and Methods

### Source of 4-Hydroxyisoleucine

4-HIL was provided by Julong Biological Engineering Co. Ltd., Henan, China.

### Animals and Diets

Male C57BL/6 mice 6–8 weeks of age were purchased from Charles River (Beijing, China). The mice were maintained using a 12 h light/dark cycle in a specific pathogen free facility with free access to food and water throughout the experiment. Animals were fed a standard laboratory chow diet (12.8% fat, 21.6% protein, 65.6% carbohydrate; HFK Bioscience, Beijing, China) or a high-fat diet (HFD, 60% fat, 26.2% protein, 26.3% carbohydrate; HFK Bioscience). The mice were randomly divided into 5 groups with eight mice in each group. One group was given standard laboratory chow diet (CHOW), and the remaining mice were given high-fat diet (HFD). After 8 weeks, the HFD group was randomly divided into HFD normal saline, HFD 4-HIL 50 mg/kg, HFD 4-HIL 100 mg/kg and HFD 4-HIL 200 mg/kg, CHOW group, which was also given normal saline. Each group was administered once a day by gavage in a volume of 0.1 ml/10 g of mouse body weight for 8 weeks. 4-HIL was dissolved into different concentrations by saline. We recorded weekly changes in body weight and blood glucose for each mouse, also the changes in water intake and food intake for each cage of mice. Animal welfare and experimental procedures were carried out in accordance with the ethical provisions on the care and use of experimental animals at Zhengzhou University and were approved by the university’s Animal Experimental Committee.

### Acute Toxicity

Twenty C57BL/6 mice with a body weight of 18–25 g, each half male and female, were adaptively fed for one week. The mice were randomly divided into a normal control group and an administration group; after fasting for 12 h, they were given normal saline by intragastric administration, Or 4-HIL (2 g/kg); 4 h after the end of the administration, freely drink and eat for 1 week, and record the weight of the mice during the breeding period to observe whether there is poisoning or death; one week later, the mice are dissected. Observe whether the main organs such as heart, liver, spleen, lung and kidney are abnormal.

### MTT

The cells were inoculated on a flat-bottomed 96-well plate at a density of 4,000 cells/well. After the cells were grown overnight, the cells were treated with serum-free medium for 12 h, and the plates were tested after 24, 48 and 72 h. 20 μL MTT was added 4 h before each detection time point to avoid light. The supernatant of the medium was discarded, 150 μL DMSO was added, and the absorbance was measured at 490 nm. The absorption values of 4-HIL at 490 nm were determined by a microplate analyzer at different concentrations of 1 μM, 10 M, 100 μM and 1,000 μM at different time points for 24, 48 and 72 h.

### Immunohistochemical Staining

Hematoxylin and eosin (HE) stain, immunohistochemistry materials, and Oil Red O stain used to examine animal tissues were all provided by Wuhan Servicebio Technology Co. Ltd. (Wuhan, China).

### Insulin Tolerance Test and Glucose Tolerance Test

To perform GTTs, the weight of each mouse in each group was measured and recorded. Each mouse was intraperitoneally injected with glucose solution (2 g/kg) and blood glucose was measured using a blood glucose meter at 0, 15, 30, 60, 90, and 120 min. The results were used to construct a curve and the area under the curve was calculated.

To perform ITTs, each mouse was intraperitoneally injected with insulin solution (0.75 U/kg) and the blood glucose of mice was measured using a blood glucose meter at 0, 15, 30, 60, 90, and 120 min. A curve was constructed and the AUC calculated. Tail blood glucose levels were monitored using a glucometer (Roche Diagnostics, Basel, Switzerland).

### Blood Serum Indices

Blood serum triglyceride (TG), total cholesterol (TC), high density lipoprotein cholesterol (HDL-C), low density lipoprotein cholesterol (LDL-C), aspartate aminotransferase (AST), and alanine aminotransferase (ALT) were determined using commercial kits (Jiancheng, Nanjing, China).

### Extraction of RNA and Quantitative Real-Time Polymerase Chain Reaction

According to the trade description, total RNA was extracted from mouse tissues using TRIZol reagent (Thermo Fisher Scientific, Waltham, MA, United States) and reverse transcribed with RT2 first chain kit (Thermo Fisher Scientific). PCR was performed using a LightCycler480 real-time PCR system (Roche Diagnostics). The 2^ΔΔCt^ method was used to calculate the multiple changes of gene expression. Objective gene and internal reference gene 36B4 primers were synthesized by Jin Weizhi Biotechnology Co., Ltd. (Beijing, China). The primer sequences used in this experiment are summarized in Supplementary Table S1.

### Western Blot Analysis

Antibodies against IkB-α (nuclear factor of kappa light polypeptide gene enhancer in B cells inhibitor alpha; 1:1,000), JNK (c-Jun N-terminal), phosphorylated JNK (1:1,000), β-actin (1:1,000) and TLR4 (1:1,000) were purchased from Cell Signaling Technology (United States). Total protein of liver and adipose tissue was extracted by adding lytic buffer to each ice-cold sample. Proteins were isolated by sodium dodecyl sulfate-polyacrylamide gel electrophoresis (SDS-PAGE) and transferred to a polyvinylidene difluoride membrane (Millikon). Each membrane was sealed in the presence of Tris buffered saline containing Tween (TBST) containing 5% skimmed milk powder at room temperature for 1 h. Then, a defined concentration of diluted antibody was added as described by the manufacturer. The blot was incubated overnight in a flip shaking bed at 4 °C and washed six times for 5 min each time using TBST. TBST containing 5% skimmed milk powder a 1:8,000 dilution of mouse anti-rabbit antibody labeled with horseradish peroxidase (HRP) and incubated at room temperature for 2 h. The blot was washed six times for 5 min each time. An enhanced chemiluminescence (ECL) detection kit (Applygen Technologies, Beijing, China) was used to display protein bands. The protein bands were analyzed by ImageJ image analysis software (NIH, Bethesda, MD, United States).

### Flow Cytometry

Mice were killed and the liver and epididymal adipose tissues were isolated. The tissues were each ground into single cell suspension and filtered through a 70 μm cell screen. After the red blood cells were lyzed, the cells were blocked using 10% rat serum in PBS for 10 min. The M1 macrophage maker was CD45^+^F4/80^+^CD11b^+^CD11c^+^ and M2 macrophage maker was CD45^+^F4/80^+^CD11b^+^CD206^+^. Representative flow cytometry plots showing the gating scheme for adipose tissue or liver macrophages. Cells from mouse were gated on forward-and side-scatter-area (FSC-A and SSC-A, respectively). immune cells were further selected by CD45 staining. CD45^+^F4/80^+^ATMs were selected and plotted to show CD11b^+^ and CD11c^+^ (M1)/CD206^+^(M2)fluorescence. Anti-mouse CD11b-APC(1:100; eBioscience), Anti-mouse CD11c-PE (1:100; eBioscience), Anti-mouse CD206-PE (1:100; eBioscience); Anti-mouse CD45-FITC (1:100; eBioscience); Anti-mouse F4/80 PerCP-Cyanine5.5 (1:100; eBioscience); Anti-mouse F4/80 FITC(1:100; eBioscience); Anti-mouse CD11b-PE (1:100; eBioscience), Anti-mouse CD11c-APC(1:100; eBioscience), Anti-mouse CD206-APC (1:100; eBioscience); Armenian hamster IgG-PE (1:100; eBioscience); Rat IgG2α kappa -FITC (1:100; eBioscience); Rat IgG2b kappa-APC (1:100; eBioscience); Rat IgG2α kappa -APC (1:100; eBioscience); Rat IgG2α kappa -PE (1:100; eBioscience); Rat IgG2α kappa PerCP-Cyanine5.5 (1:100; eBioscience) antibodies were also used. The streaming data was obtained by using a FACS Calibur device (BD Biosciences, Santa Clara, CA, United States) and analyzed and processed by FlowJo software.

### Preparation of Mouse Bone Marrow-Derived Macrophages

Bone marrow cells were isolated from 8 to 12-week-old mice. The hind limb bones were isolated in the biosafety cabinet after the mice were killed. DMEM medium was aspirated using a 1 ml syringe to flush out the bone marrow. The single-cell suspension was collected and filtered with a sieve. The bone marrow cells were centrifuged, and 5 ml red blood cell lysate was added for lysis at room temperature for 5–8 min. After washing with PBS twice, red blood cells were removed to obtain bone marrow cells, which were used to induce macrophages. To generate M0 or M1 macrophages, sorted monocytes or bone marrow cells were treated for 7 days with 20 ng/ml of either recombinant human or mouse granulocyte macrophage colony-stimulating factor (GM-CSF). M1 polarization was achieved on Day 5 by stimulation with 20 ng/ml of interferon gamma (IFN-γ) for 1 h, followed by 100 ng/ml lipopolysaccharide (LPS) for 48 h. To generate M2 macrophages, cells were cultured in the presence of M-CSF (20 ng/ml) for 7 days, at the 6 and 7 days IL-13 (20 ng/ml), IL4 (20 ng/ml) were add. The cells were inoculated into wells of a 6-well plate at the density of 2 × 10^5^/well in a 6-well plate. The cells were incubated overnight (12 h) in serum-free DMEM. After this period of synchronization, 4-HIL was added to the culture for 24 h. Control cells were not treated. Both groups of macrophages were examined by flow cytometry.

### Statistical Analysis

The data are shown as means ± s.e.m. Data sets that involved more than two groups were assessed by one-way ANOVA followed by Newman–Keuls post hoc tests. Next generation sequencing analysis was assessed using Tukey’s honest significant difference post hoc tests. In the figures, the data with *are different based on post hoc ANOVA statistical analysis. SPSS version 20.0 was used. The statistical analysis diagram is completed by software GraphPad Prism8. *p < 0.05; ** 0.001 < *p* < 0.01; or ***p < 0.001.

## Results

### 4-Hydroxyisoleucine Decreases Body Weight and Hyperglycemia of Obese Mice Induced by High-Fat Diet

**FIGURE 1:**
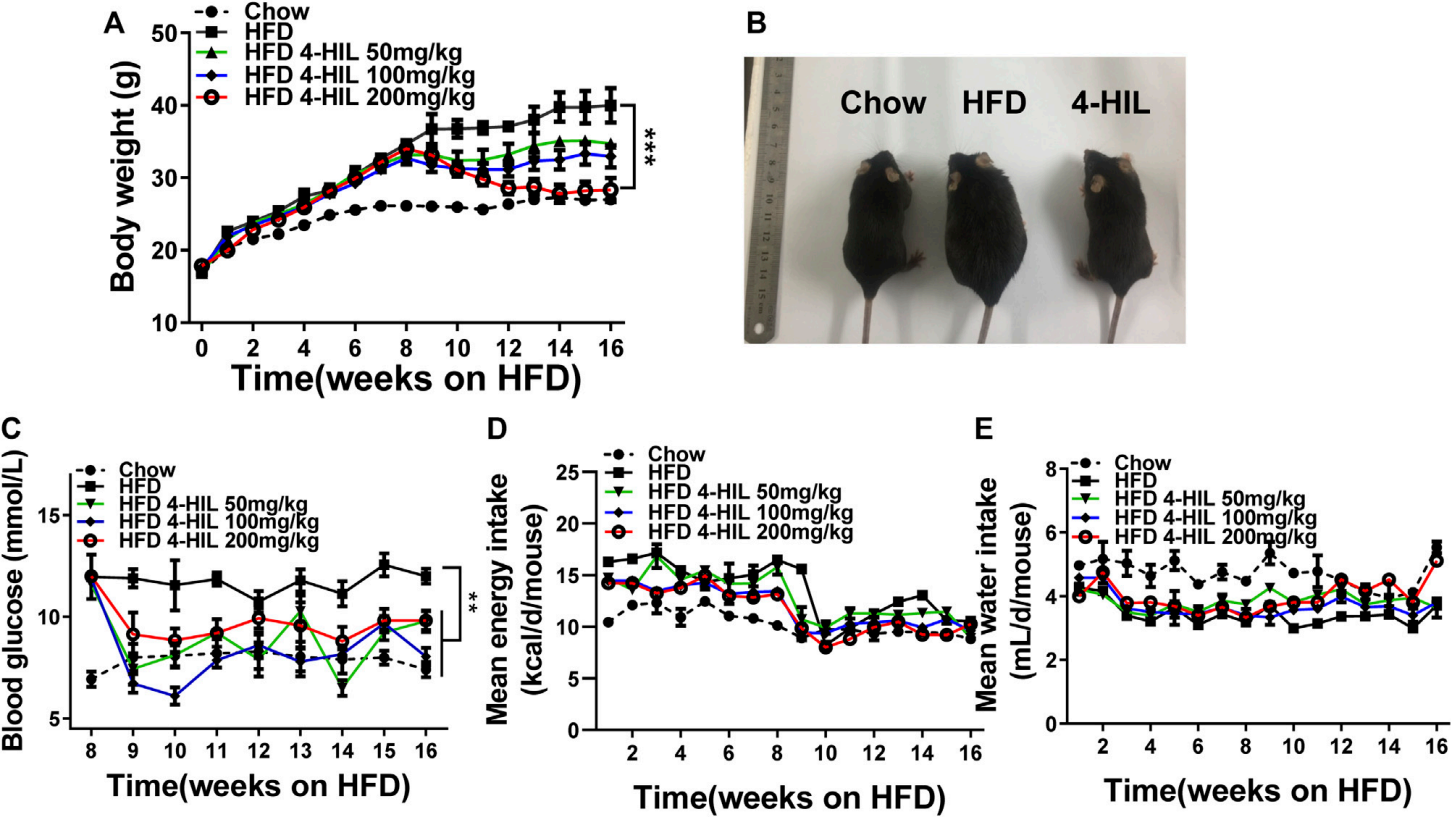
4-HIL therapy reverses HFD-induced obesity and hyperglycemia. Mice were fed with HFD for 16 weeks and body weight **(A)**, diet consumption **(D)**, and drinking water consumption **(E)** were recorded every week. 4-HIL intervention was given every day from week 9 and the change of blood glucose was detected every week after administration **(C)**. **(B)** Representative photographs of mice in the CHOW group **(left)**, HFD group **(middle)**, and 4-HIL (200 mg/kg) group **(right)**. *, 0.01 < *p* < 0.05; **, 0.001 < *p* < 0.01; ***, *p* < 0.001 (*n* = 8 for each group).

C57BL/6 mice were fed the HFD for 8 weeks, and then fed HFD supplemented with 4-HIL (50, 100, or 200 mg/kg) for another 8 weeks. Mice fed HFD 16 weeks became obese and developed hepatic steatosis, hyperlipidemia, and insulin resistance. The body weight, diet, and drinking water consumption of each mouse was measured and recorded weekly. Blood sugar was monitored weekly after drug intervention. 4-HIL lessened body weight and diet-related obesity. The 8-weeks treatment with the different doses of 4-HIL significantly decreases the body weights of mice in a dose-dependent manner. Use of 200 mg/kg 4-HIL lowered body weight of mice to almost the same weight as the control mice (Figures 1A,B). The intervention of 4-HIL had no effect on the diet consumption and water intake of mice (Figures 1D,E), indicating that the weight loss of mice after administration was not caused by the reduced consumption of food or drinking water. Monitoring of blood glucose levels in mice after administration of 4-HIL revealed reduced blood glucose levels (Figure 1C).

### 4-Hydroxyisoleucine Markedly Improves Insulin Sensitivity and Reverses Insulin Resistance

**FIGURE 2:**
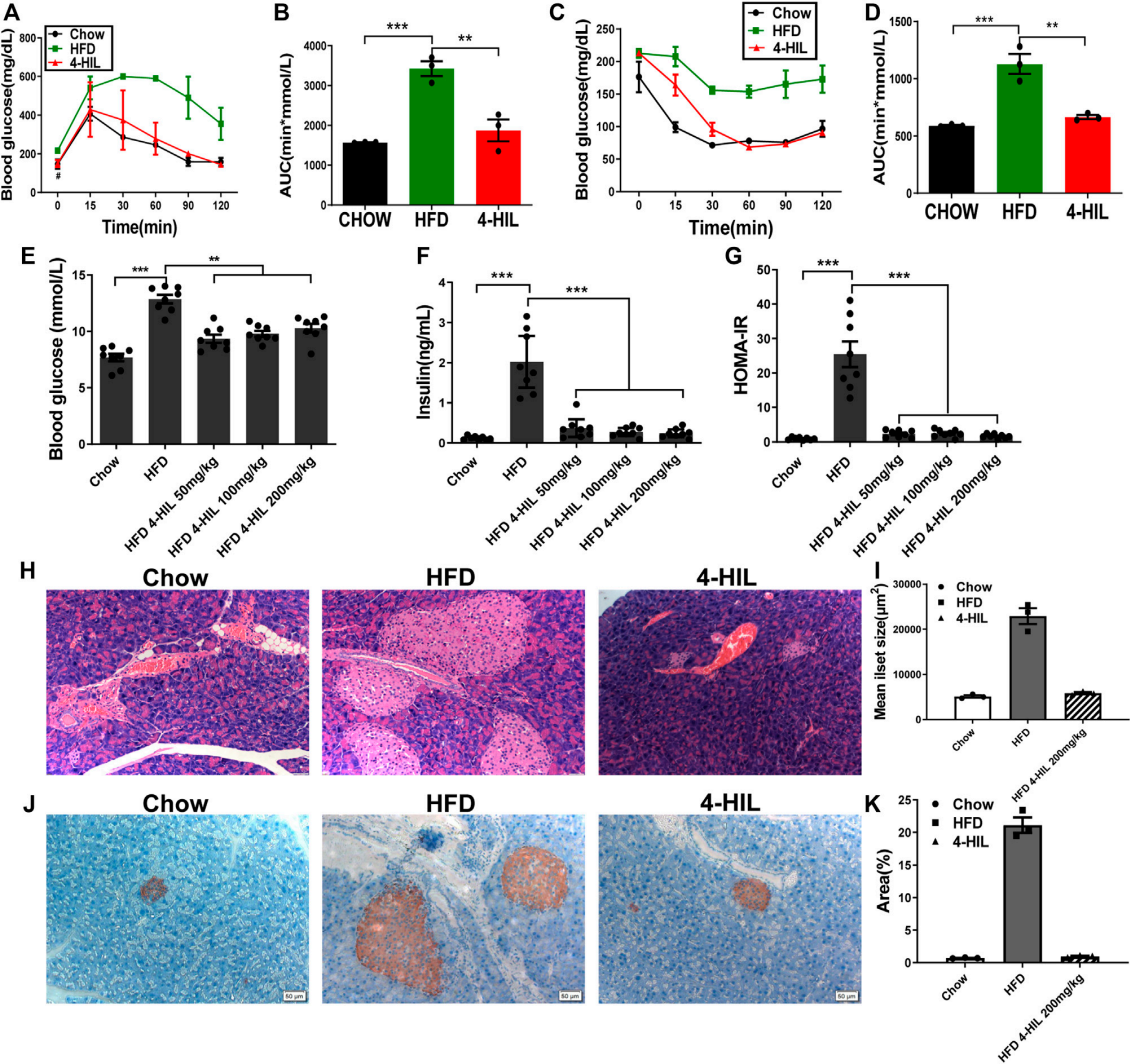
4-HIL improves insulin sensitivity. After 4-HIL administration, the mice in CHOW, HFD, and 4-HIL (200 mg/kg) were fasted for 8 h prior to glucose tolerance test **(A)** and insulin tolerance test **(C)**. The area under the curve **(B)**, **(D)** was calculated. After fasting for 8 h, blood samples of mice were taken from the tail tip to measure blood glucose using a blood glucose meter **(E)**, and the concentration of insulin in the sample was measured by ELISA **(F)**. The insulin resistance index **(G)** was calculated according to the formula provided in the text. At the end of administration, the isolated pancreatic tissues of mice were used for HE staining **(H)** and insulin antibody immunohistochemical staining **(J)**. The mean islet area **(I)** and insulin positive **(K)** were analyzed and quantified using ImageJ software. *, 0.01 < *p* < 0.05; **, 0.001 < *p* < 0.01; ***, *p* < 0.001 (*n* = 8 for each group).

To verify the effect of the drug on insulin resistance, the GTT was used to detect glucose tolerance in mice. Due to the long-term intake of high glucose and high-fat, the blood glucose of the HFD group increased sharply after injection of glucose and did not recover within 2 h. Mice in the HFD group had abnormal glucose metabolism and glucose intolerance (Figures 2A,B). The glucose tolerance in the 4-HIL treatment group was significantly improved compared with the HFD control group, and the area under the curve was significantly different from that in the model control group (*p* < 0.05). The ITT results showed that the model control group was insensitive to insulin and the hypoglycemic ability decreased after injection of insulin. The hypoglycemic ability of 4-HIL group was significantly higher than that of HFD group, and decreased significantly below the curve (*p* < 0.001), indicating that 4-HIL increased insulin sensitivity mi the mice (Figures 2C,D). After 8 weeks of administration, blood samples were collected from the tail tip of each mouse to measure blood glucose, blood samples were taken from the orbit of the eye to obtain plasma, and plasma insulin concentration was measured by the double antibody sandwich ELISA method. Compared with the CHOW group, the fasting plasma insulin content in the HFD group was significantly higher than that in the 4-HIL group (*p* < 0.01), and the 4-HIL intervention group significantly reversed the increase in fasting plasma insulin content in mice (Figures 2E,F). In the model control group, due to the intake of high-sugar and HFD, the increase of blood glucose led to the compensatory increase of insulin secretion, which led to the compensatory increase of fasting plasma insulin content in the mice. The 4-HIL treatment group displayed significant improvement that was dose-dependent. We calculated the insulin resistance index (fasting blood glucose level (mmol/L) × fasting insulin level (mIU/L)/22.5). Treatment of 4-HIL significantly decreased the insulin resistance index (*p* < 0.05, Figure 2G).

Insulin is the only hormone in the body that lowers blood sugar. In the early stage, we found that the insulin sensitivity was improved after 4-HIL treatment. Furthermore, the effect of 4-HIL on insulin-secreting pancreatic tissue was verified by HE staining, and the average islet area was calculated by ImageJ software. The islet area increased significantly in the model control group, but decreased significantly in the 4-HIL group (Figures 2H,I). Insulin antibody was used to detect islet *β* cells, and the results were analyzed and quantified by ImageJ software. A large proportion of the cytoplasm in the islet cells were positive, but there was no obvious positive reaction in the extra islet acinar cells, and the positive rate in the model control group was significantly higher than that in the normal control group (Figures 2J,K). These results confirmed our hypothesis that, due to the decreased insulin sensitivity in the model control group, the intake of high-sugar and HFD stimulates the compensatory secretion of insulin by islet *β* cells to regulate blood glucose, resulting in compensatory proliferation of islet *β* cells. The intervention of 4-HIL significantly inhibited the compensatory proliferation of islet *β* cells, which further indicated that insulin sensitivity was improved after administration of 4-HIL.

### 4-Hydroxyisoleucine Improves Dyslipidemia and Reduces Lipid Ectopic Accumulation in Model Mice

**FIGURE 3:**
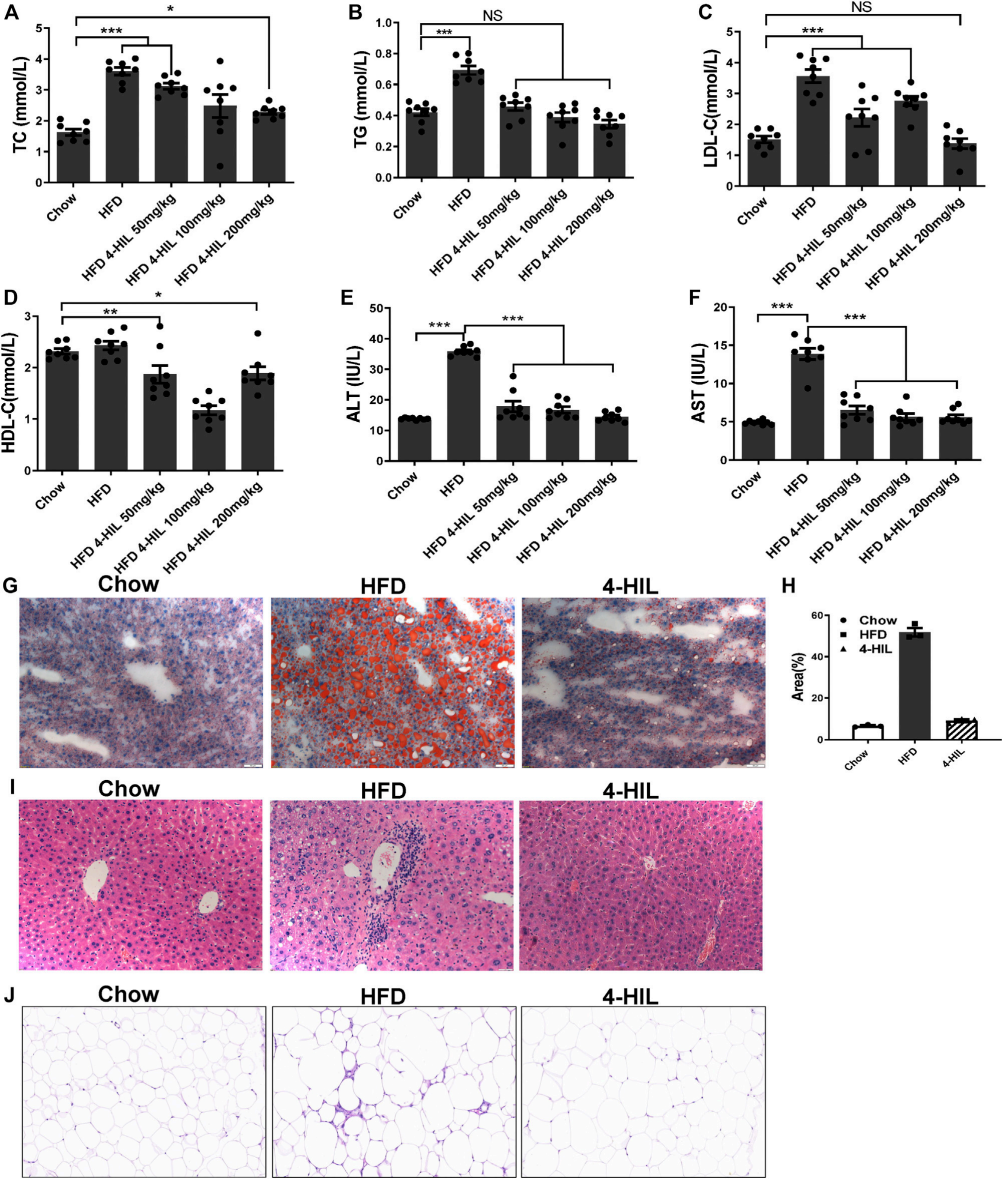
4-HIL reduces dyslipidemia. After 4-HIL administration, blood samples were taken from the orbit of mice after fasting for 8 h, and the contents of TC **(A)**, TG **(B)**, LDL-C **(C)**, HDL-C **(D)**, ALT **(E)**, and AST **(F)** in plasma were measured according to the instructions of the kits. Oil Red O staining of liver tissue (200×) **(G)**, HE staining of liver tissue (200×) **(I)**, and HE staining of adipose tissue (200×) **(J)** are presented. *, 0.01 < *p* < 0.05; **, 0.001 < *p* < 0.01; ***, *p* < 0.001 (*n* = 8 for each group).

After 16 weeks of the HFD, the liver weight and especially fat accumulation of mice had increased significantly. The liver weight and fat accumulation were significantly lower in the 4-HIL treatment group as compared with the HFD group. The decrease was related to the 4-HIL dose and had no significant effect on the kidney (Table 1). In order to explore the effect of 4-HIL on blood lipids in the HFD-related obese mice, we used the isolated plasma to determine the four contents of blood lipids. Dyslipidemia occurred in the HFD group, with reduced levels of blood lipid evident in the 4-HIL treatment group (Figures 3A–D). HE staining of liver tissue showed that after induction by high glucose and high-fat, mouse liver cells contained many fat vacuoles in mouse liver cells, mainly small and medium-sized vacuoles, accompanied by inflammatory cell infiltration, consistent with reports in the literature (Gregor and Hotamisligil, 2011; Saltiel and Olefsky, 2017). In the 4-HIL group, the degree of vesicular degeneration and steatosis of liver cells was significantly reduced, and the number of fat vacuoles was significantly reduced (Figure 3I). After Oil Red O staining, the number and volume of fat droplets in liver cells of mice in the 4-HIL group were significantly lower than those in HFD group (Figures 3G,H). HE staining of epididymal adipose tissue revealed that the adipocytes in the model control group were significantly hypertrophic and the inflammatory infiltration of the crown-like structure was obvious, consistent with reports in the literature (Han and Levings, 2013; Lee et al., 2013; Tanaka et al., 2014). The volume of adipocytes and inflammatory infiltrating cells decreased significantly in the 4-HIL group, which indicated that 4-HIL could inhibit adipocyte hypertrophy and reduce inflammatory infiltration (Figure 3J). The long-term intake of the high-sugar and HFD damaged liver function, as evident by the significant increases in plasma AST and ALT. 4-HIL significantly reduced the increase plasma AST and ALT caused by HFD (Figures 3E,F).

**TABLE 1:** Body and organ weights at the end of treatment.

	CHOW	HFD	4-HIL		
			50 mg/kg	100 mg/kg	200 mg/kg
Liver weight (g)	0.90 ± 0.03	1.29 ± 0.22	1.08 ± 0.03	0.99 ± 0.06	0.99 ± 0.12*
Kidney weight (g)	0.30 ± 0.01	0.39 ± 0.03	0.38 ± 0.02	0.36 ± 0.03	0.33 ± 0.04
Fat pad (g)	0.61 ± 0.08	7.36 ± 0.99	3.52 ± 0.5	3.14 ± 0.73	1.51 ± 0.47*
Liver index (%)	3.38 ± 0.08	3.00 ± 0.35	3.18 ± 0.06	3.18 ± 0.24	3.42 ± 0.20
Kidney index (%)	1.13 ± 0.02	0.91 ± 0.02	1.12 ± 0.03	1.13 ± 0.03	1.15 ± 0.06
Index of fat (%)	2.28 ± 0.28	17.10 ± 1.25	10.12 ± 1.2	9.14 ± 1.84	5.13 ± 1.32**

Data were analyzed using one-way ANOVA. Values (mean ± SEM; *n* = 8) were taken at the end of the treatment day.

### 4-Hydroxyisoleucine Reduces Expression of Proinflammatory Cytokines

**FIGURE 4:**
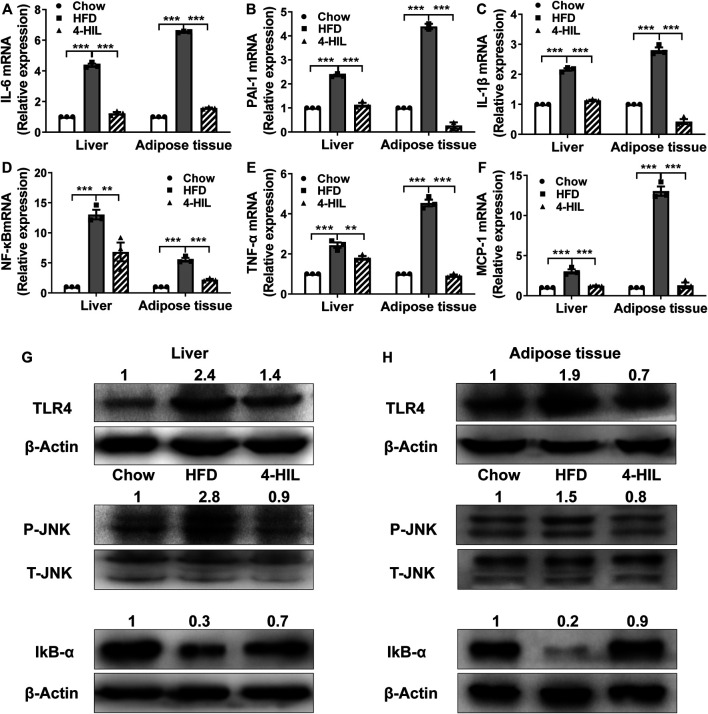
4-HIL decreases proinflammatory cytokine expression. After 8 weeks of 4-HIL (200 mg/kg)administration, the mRNA expression levels of IL-6 **(A)**, PAI-1 **(B)**, IL-1β **(C)**, NF-κB **(D)**, TNF-α **(E)**, and MCP-1 **(F)** in mouse liver and adipose tissues were detected by qRT-PCR. The relative level of mRNA was determined by comparing the relative expression of mRNA with that of the CHOW group by the Ct method. Effect of 4-HIL (200 mg/kg) on TLR4 related signaling pathway **(G)–(H)**. Representative immunoblotting of target proteins was used to detect the effects of 4-HIL treatment on TLR4 protein production, JNK phosphorylation and IkB-α production in liver and epididymal adipose tissues. *, 0.01 < *p* < 0.05; **, 0.001 < *p* < 0.01; ***, *p* < 0.001 (*n* = 3 for each group).

In order to further study the effect of 4-HIL on obesity-related chronic inflammation, we measured the levels of inflammatory gene mRNA in liver and adipose tissue (Figures 4A–F). The expression levels of tumor necrosis factor-alpha (TNF-α), IL-1 β, IL-6, plasminogen activator inhibitor-1 (PAI-1), monocyte chemoattractant protein-1 (MCP-1), and NF- κ B in liver and adipose tissue of the model control group were significantly increased as compared with the values reported in the literature (Chawla et al., 2011). However, the expression of inflammatory gene mRNA decreased significantly after 4-HIL intervention. The Toll-like receptor 4 (TLR4) signaling pathway induces the production of proinflammatory cytokines by regulating the activities of c-Jun N-terminal kinase (JNK) and NF-κB, and leads to chronic inflammation and insulin resistance. TLR4 knockout mice fed with a high-sugar and HFD did not develop obesity and insulin resistance (Shi et al., 2006; Jia et al., 2014; Wada and Makino, 2016). We detected TLR4-related signaling pathways in liver and adipose tissue by western blot. Compared with HFD group, 4-HIL reduced the expression of TLR4 protein and inhibited JNK phosphorylation in liver and adipose tissue of HFD mice. Increasing the production of nuclear factor of kappa light polypeptide gene enhancer in B-cells inhibitor, alpha (IκB-α) in NF-κ B interaction can prevent the translocation and activation of NF-κ B. The 4-HIL intervention decreased the expression of TLR4, inhibited the phosphorylation of JNK, and increased the production of IκB-α (Figures 4G,H).

### 4-Hydroxyisoleucine Reduces the Proportion of Proinflammatory M1 Macrophages

**FIGURE 5:**
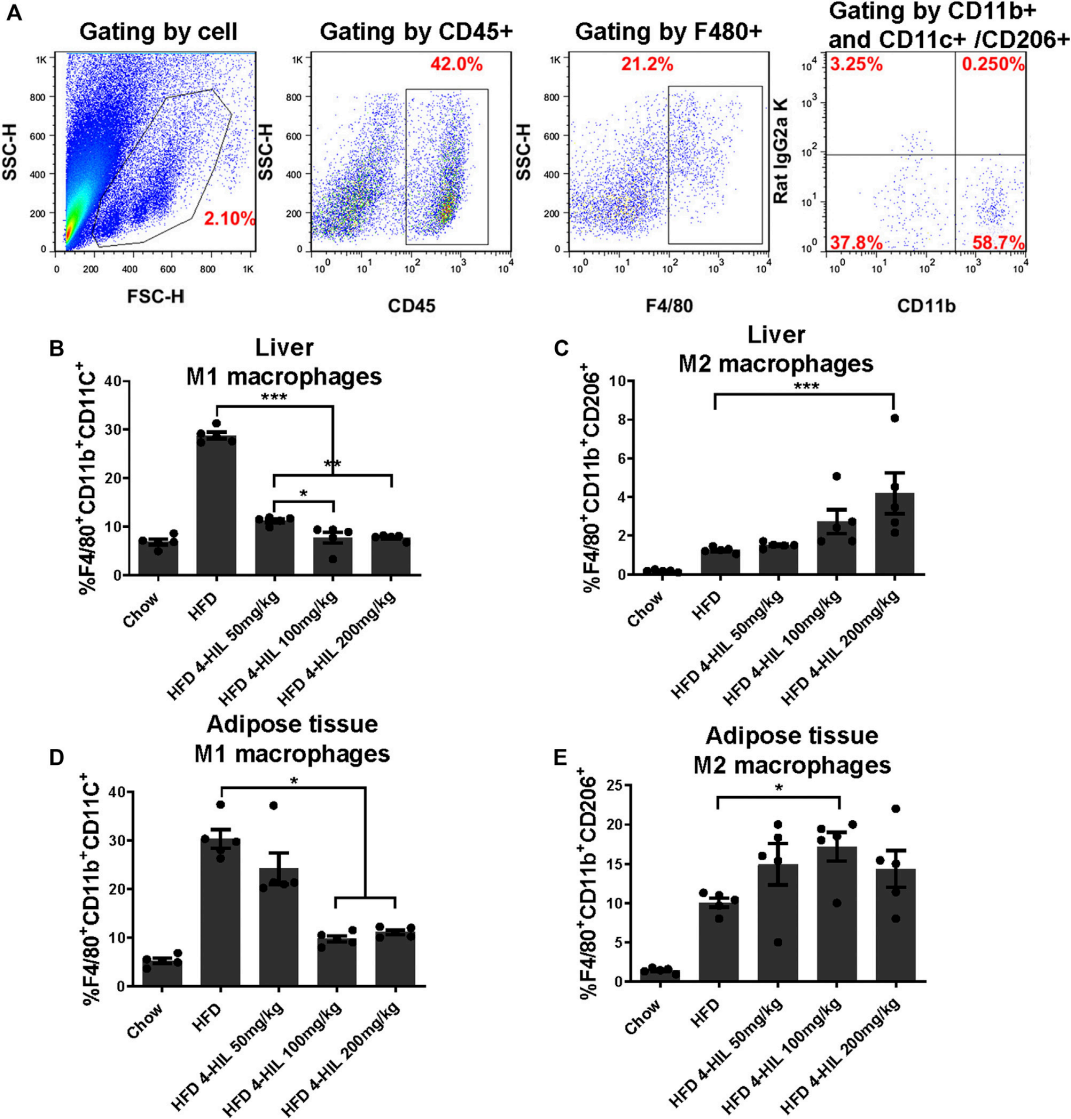
Proinflammatory M-1-like CD11c^+^ macrophages from HFD-fed mice are inhibited by 4-HIL *in vivo*. After the end of the period of 4-HIL administration, the liver and adipose tissue were obtained and the tissues was processed to single cell suspensions. The antibody was added and incubated in the dark, and the effect of the 4-HIL administration on the macrophage was detected by flow cytometry. Typical flow cytometry shows the gating scheme of macrophages **(A)**. Fluorescence-activated cell sorting gating results of macrophage flow cytometry are shown in **(B)**–**(E)**.

**FIGURE 6:**
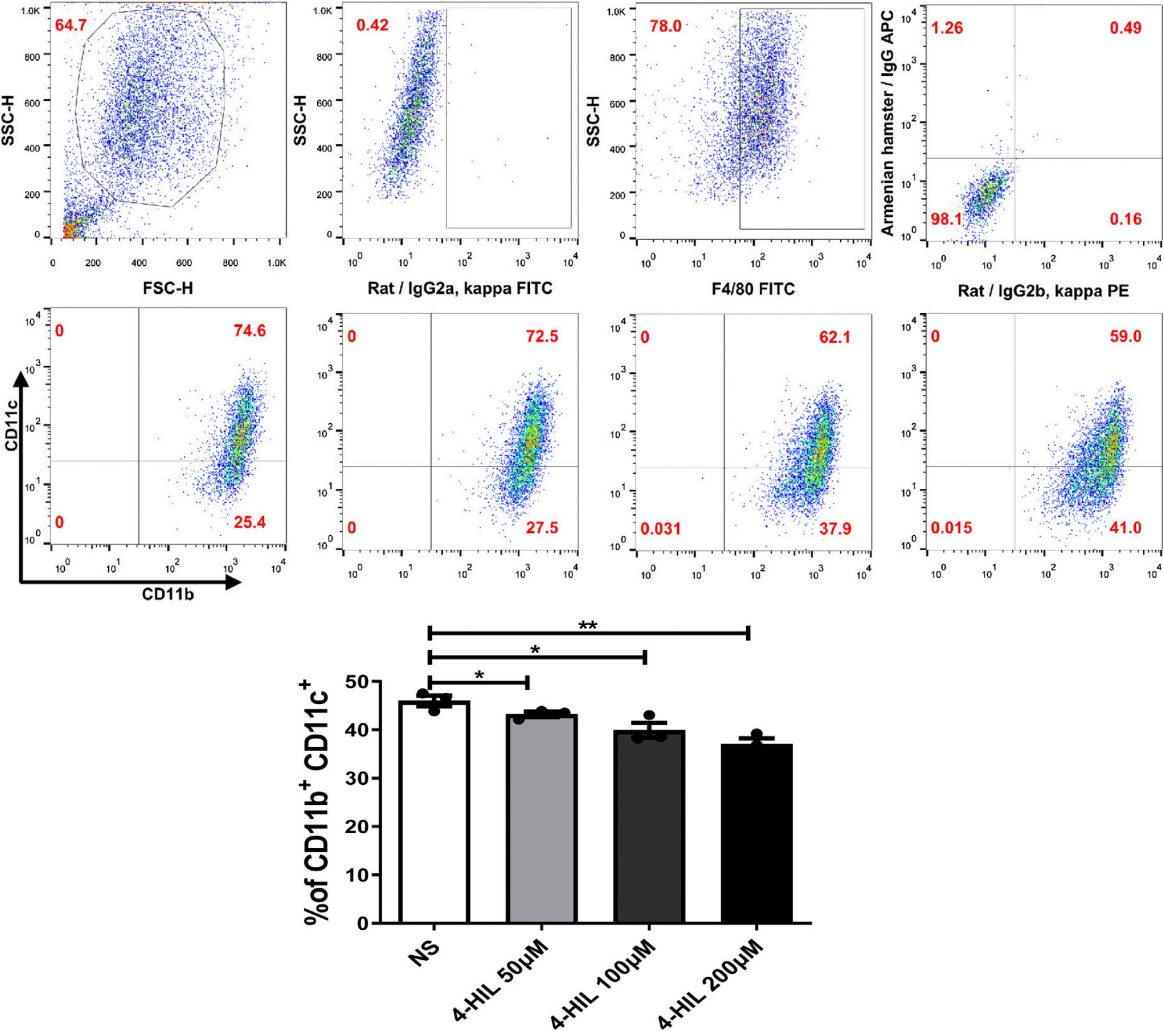
Effect of 4-HIL on macrophages *in vitro*. Bone marrow-derived cells were cultured with GM-CSF (20 ng/ml) for 7 days. INF- γ (20 ng/ml) and LPS (100 ng/ml) was added on the day 6 and 7, respectively. After removing the non-adherent cells, the adherent cells were exposed to 4-HIL for 24 h. The proportion of macrophages was analyzed by flow cytometry.

Macrophage recruitment and polarization play key roles in obesity-related chronic inflammation. In order to study the effect of 4-HIL on macrophages in liver and adipose tissue, the proportion of macrophages in liver tissue and adipose tissue was analyzed by fluorescence-activated cell sorting. The proportion of M1 macrophages in liver tissue and adipose tissue of mice in the HFD group was higher than that in normal control group. The level of proinflammatory macrophages in mice treated with 4-HIL decreased in a dose-dependent manner. The 4-HIL treatment reduced the number of M1 macrophages in the liver and adipose tissue of HFD mice, and significantly reduced the increase of M1-ATM and the decrease of M2-ATM induced by HFD (Figures 5A–E). These findings suggested that the inflammatory state was reduced, consistent with the observed decrease in the expression of a variety of proinflammatory genes in adipose tissue and liver tissue after 4-HIL administration. Flow cytometry analysis revealed that 4-HIL administration reduced the proportion of M1 macrophages in liver and adipose tissue, and also reduced the high expression of MCP-1 induced by HFD at the mRNA level. We further explored whether 4-HIL could directly affect the polarization of macrophages and thus, reduce inflammation and improve metabolism-related diseases. We induced mouse BMDM *in vitro* to observe whether 4-HIL can directly affect M1 macrophages. After macrophages induced *in vitro* were treated with 4-HIL for 24 h, the proportion of M1 macrophages was detected by flow cytometry. Administration of 4-HIL in the macrophage model *in vitro* could still reduce the proportion of M1 macrophages. However, there was no difference in the proportion of M2 macrophages (Figures 6A–C).

## Discussion

One of the important findings of this study is that 4-HIL intervention can led to significantly weight reduction. In particular, even if the HFD was continued for 8 weeks, the concurrent use of 4-HIL delayed obesity, steatosis, hyperglycemia and insulin resistance in the obese mice. There was no significant difference in daily food intake between HFD and 4-HIL groups, suggesting that 4-HIL attenuated HFD-induced obesity through metabolic regulation. The findings demonstrate that 4-HIL improves metabolic disorders and reduces obesity-related inflammation in HFD mice by reducing the proportion of M1 macrophages and inhibiting the function of M1 macrophages, thereby inhibiting chronic inflammation in adipose and liver tissues. Furthermore, 4-HIL significantly increased insulin sensitivity of and improved the dysfunctions in glucose and lipid metabolism in the model mice.

According to the literature, 4-HIL can stimulate the insulin secretion of isolated rat pancreas (Sergent et al., 2008). Our experimental results showed that the regulation of blood glucose level by 4-HIL occurred mainly through the increase of insulin sensitivity, but not increased insulin secretion. In order to verify whether 4-HIL increased insulin secretion *in vivo*, GTT was performed in normal C57BL/6 mice to evaluate whether the drug stimulated insulin secretion. The blood glucose level in mice administered 4-HIL at 200 mg/kg for 15 min group was significantly lower than that in the vehicle group, and other time points had no significant effect on blood glucose value and AUC value (Supplementary Figure S1). We also applied 4-HIL to epinephrine-induced hyperglycemia mice and observed that 4-HIL had no significant hypoglycemic effect on subacute epinephrine hyperglycemia model (Supplementary Figure S2). However, in the HFD model, 4-HIL could significantly reduce the blood glucose value and AUC value, the serum insulin content in the 4-HIL treatment group was significantly lower than that in the HFD group, and the Homeostatic Model Assessment of Insulin Resistance value showed that the insulin resistance in the treatment group was significantly decreased. The results of HE staining and insulin antibody immunohistochemistry showed that the area of insulin area (β cell dense area) in the HFD group was significantly increased because high glucose and high lipid intake induced insulin resistance and led to compensatory proliferation of islet β cells. Islet β cells secrete a large amount of insulin in a compensatory response to regulate blood glucose. The significant reduction in the islet area of the 4-HIL intervention group further proved that the insulin content of mice in the treatment group was decreased.

Obese mice produce high levels of proinflammatory cytokines, including TNF- α, IL-1 β, IL-6, and PAI-1, in liver and adipose tissue, and obesity is characterized by infiltration and activation of immune cells in liver and adipose tissues (Chawla et al., 2011; Bu et al., 2018). The expressions of inflammatory gene mRNAs in the 4-HIL treatment group were significantly decreased, indicating that 4-HIL can reduce the level of inflammatory factors in insulin target organs of HFD model mice. TLR4 signal leads to the production of proinflammatory cytokines in the target tissue of HFD mice and leads to chronic inflammation and insulin resistance (Shi et al., 2006; Cani et al., 2008). The intervention of 4-HIL decreased the expression of TLR4, inhibited the phosphorylation of JNK, and increased the production of IκB-α. These results indicated that the administration of 4-HIL could significantly improve the state of chronic inflammation in the body. 4-HIL can reduce the ratio of M1/M2 in liver and adipose tissues of HDF mice, affect the polarization of macrophages, and improve the chronic inflammatory response. Flow cytometry examination of liver and adipose tissues of HDF mice after 4-HIL administration revealed the accumulation of a large number of proinflammatory macrophages, consistent with prior observations in the literature (Pal et al., 2012). The level of proinflammatory macrophages in 4-HIL treatment group decreased in a dose-dependent manner. We further explored *in vitro* whether 4-HIL directly affects the polarization of macrophages, and observe the direct effect of 4-HIL on macrophages by inducing mouse BMDM. 4-HIL could still reduce the proportion of M1 macrophages *in vitro*, and similar results were found in both BMDM and M1 macrophage models *in vitro*. At the same time, we also studied the effect of 4-HIL on BMDM induced in TLR4 deficient mice. The proportion of M1 macrophages in these mice was still reduced, indicating that the effect of 4-HIL does not depend on TLR4.

Obesity, especially the increase of visceral fat content, will lead to the occurrence and development of metabolism-related diseases (Moller and Kaufman, 2005). The increase of free fatty acids and lipid accumulation in liver and other organs are the main causes of insulin resistance. Studies in humans and mice have shown that the size of adipocytes is a powerful predictor of the proportion of macrophages in adipose tissue (Weisberg et al., 2003). Adipocyte volume is closely associated with systemic insulin resistance, dyslipidemia, and the risk of type 2 diabetes, and weight loss is accompanied by a decrease in adipocyte volume. The liver is an important site of lipid metabolism. In the HFD group, the liver was light yellow and had a tight capsule, blunt edge, slightly soft texture, and greasy touch. Oil Red O staining revealed the pronounced accumulation of lipids, accompanied by a significant increase in plasma AST and ALT levels. This is consistent with literature reports. As the site of crosstalk between adipocytes and macrophages, there is a unique structure in obese adipose tissue called crown-like structure (CLS), where macrophages are considered to scavenge the residual lipid droplets of dead adipocytes (Cinti et al., 2005; Lumeng et al., 2007). Histologically, proinflammatory M1 macrophages aggregate to constitute CLS in obese adipose tissue of humans and rodents. On the other hand, M2 macrophages are scattered in the interstitial spaces between adipocytes. Notably, the number of CLS is positively correlated with systemic insulin resistance in obese subjects (Apovian et al., 2008; Bremer et al., 2011), suggesting the pathophysiologic role of CLS in adipose tissue inflammation and systemic energy metabolism (Tanaka et al., 2014). In the 4-HIL group, the amount of fat accumulation was significantly decreased, and serum AST and ALT were significantly decreased. Tissue sections showed that the number of adipocytes decreased, the cell volume significantly decreased, and the infiltration of inflammatory cells decreased, indicating that 4-HIL can improve the disorder of lipid metabolism caused by high-fat. Weisberg et al. reported that the size of adipocytes is related to the number of macrophages in adipose tissue (Weisberg et al., 2003), and the regulation of adipose metabolism by 4-HIL is closely related to macrophages.

These results were derived from a diet-induced obesity model in mice. Whether 4-HIL has a similar effect on insulin resistance in patients with type 2 diabetes remains to be studied. The activation characteristics of mouse macrophages and human macrophages are different (Mantovani et al., 2004). For example, the activity of inducible nitric oxide synthase in macrophages is different between mouse and human inflammatory models (Zhang et al., 1996; Schneemann and Schoedon, 2002). However, for many inflammatory markers, obese human and mouse adipose tissue macrophage (ATM) are similar, so mice are still useful models for testing the biology of ATM (Weisberg et al., 2003; Xu et al., 2003; Cancello et al., 2005). Future studies will focus on the importance of identified genes for ATM function and the development of type 2 diabetes. In addition, we found that 4-HIL significantly reduced fat accumulation in obese model animals, but had no significant effect on diet. These findings need to be assessed in more depth. Whether 4-HIL can produce heat by blocking the function of the RCAN1 gene or whether it promotes the conversion of white fat into brown fat, individuals can maintain low body fat levels without the need to reduce food intake or increase exercise (Bal et al., 2012). Alternately, as reported by Eichmann et al., by reducing fat absorption, excess fat in the intestinal cavity with fecal excretion may occur (Zhang et al., 2018). These aspects still need to be investigated.

## Conclusion

In conclusion, 4-HIL significantly improved glucose and lipid metabolic dysfunctions in HFD mice. 4-HIL reduced the proportion of M1 macrophages in liver and adipose tissues of mice. By inhibiting the aggregation of M1 macrophages and inhibiting the chronic inflammation of adipose tissue and liver mediated by inflammation, metabolic disorders and chronic inflammation could be improved, and the insulin sensitivity could be increased. 4-HIL displayed no obvious toxic and side effects *in vitro* and *in vivo*. 4-HIL is a potential drug for the treatment of obesity-related metabolic diseases.

## Data Availability Statement

The raw data supporting the conclusions of this article will be made available by the authors, without undue reservation, to any qualified researcher.

## Ethics Statement

The animal study was reviewed and approved by Animal Experimental Committee of Zhengzhou University.

## Author Contributions

GL conceived and designed the experiments. JY performed the experiments. PZ, YR and YY helped to perform the experiments. GL, JY, YQ and YG analyzed and interpreted the results. JY, SS, YW, GL, YQ and YG wrote the manuscript with inputs from all authors. GL is the guarantor of this work, so he has full access to all the data in the study and is responsible for the completeness of the data and the accuracy of the data analysis. All authors discussed the results and gave final approval of the manuscript.

## Funding

Support for the studies described here was provided by the Natural Science Foundation of China (81571547, 81601448) and the Henan Province and the Key Scientific Research Projects of Henan Higher Education Institutions (19A180007).

## Conflict of Interest

The authors declare that the research was conducted in the absence of any commercial or financial relationships that could be construed as a potential conflict of interest.

## References

[B1] Apovian C. M. Bigornia S. Mott M. Meyers M. R. Ulloor J. Gagua M. (2008). Adipose macrophage infiltration is associated with insulin resistance and vascular endothelial dysfunction in obese subjects. Arterioscler. Thromb. Vasc. Biol. 28, 1654–1659. 10.1161/ATVBAHA.108.170316 18566296 PMC2728436

[B2] Bal N. C. Maurya S. K. Sopariwala D. H. Sahoo S. K. Gupta S. C. Shaikh S. A. (2012). Sarcolipin is a newly identified regulator of muscle-based thermogenesis in mammals. Nat. Med. 18, 1575–1579. 10.1038/nm.2897 22961106 PMC3676351

[B3] Bijnen M. Josefs T. Cuijpers I. Maalsen C. J. Van De Gaar J. Vroomen M. (2018). Adipose tissue macrophages induce hepatic neutrophil recruitment and macrophage accumulation in mice. Gut. 67, 1317–1327. 10.1136/gutjnl-2016-313654 29074725

[B4] Bremer A. A. Devaraj S. Afify A. Jialal I. (2011). Adipose tissue dysregulation in patients with metabolic syndrome. J. Clin. Endocrinol. Metab. 96, E1782–E1788. 10.1210/jc.2011-1577 21865369 PMC3205887

[B5] Broca C. Gross R. Petit P. Sauvaire Y. Manteghetti M. Tournier M. (1999). 4-Hydroxyisoleucine: experimental evidence of its insulinotropic and antidiabetic properties. Am. J. Physiol. 277, E617–E623. 10.1152/ajpendo.1999.277.4.E617 10516120

[B6] Broca C. Manteghetti M. Gross R. Baissac Y. Jacob M. Petit P. (2000). 4-Hydroxyisoleucine: effects of synthetic and natural analogues on insulin secretion. Eur. J. Pharmacol. 390, 339–345. 10.1016/s0014-2999(00)00030-3 10708743

[B7] Bu Y. Okunishi K. Yogosawa S. Mizuno K. Irudayam M. J. Brown C. W. (2018). Insulin regulates lipolysis and fat mass by upregulating growth/differentiation factor 3 in adipose tissue macrophages. Diabetes 67, 1761–1772. 10.2337/db17-1201 29945891

[B8] Cancello R. Henegar C. Viguerie N. Taleb S. Poitou C. Rouault C. (2005). Reduction of macrophage infiltration and chemoattractant gene expression changes in white adipose tissue of morbidly obese subjects after surgery-induced weight loss. Diabetes 54, 2277–2286. 10.2337/diabetes.54.8.2277 16046292

[B9] Cani P. D. Bibiloni R. Knauf C. Waget A. Neyrinck A. M. Delzenne N. M. (2008). Changes in gut microbiota control metabolic endotoxemia-induced inflammation in high-fat diet-induced obesity and diabetes in mice. Diabetes 57, 1470–1481. 10.2337/db07-1403 18305141

[B10] Chawla A. Nguyen K. D. Goh Y. P. (2011). Macrophage-mediated inflammation in metabolic disease. Nat. Rev. Immunol. 11, 738–749. 10.1038/nri3071 21984069 PMC3383854

[B11] Cinti S. Mitchell G. Barbatelli G. Murano I. Ceresi E. Faloia E. (2005). Adipocyte death defines macrophage localization and function in adipose tissue of obese mice and humans. J. Lipid Res. 46, 2347–2355. 10.1194/jlr.M500294-JLR200 16150820

[B12] Fowden L. Pratt H. M. Smith A. (1973). 4-Hydroxyisoleucine from seed of *Trigonella foenum-graecum* . Phytochemistry 12, 1707–1711. 10.1016/0031-9422(73)80391-7

[B13] Gregor M. F. Hotamisligil G. S. (2011). Inflammatory mechanisms in obesity. Annu. Rev. Immunol. 29, 415–445. 10.1146/annurev-immunol-031210-101322 21219177

[B14] Haiyan X. Barnes G. T. Qing Y. Guo T. Daseng Y. Chou C. J. (2003). Chronic inflammation in fat plays a crucial role in the development of obesity-related insulin resistance. J. Clin. Invest. 112 (12), 1821–1830. 10.1172/JCI19451 14679177 PMC296998

[B15] Han J. M. Levings M. K. (2013). Immune regulation in obesity-associated adipose inflammation. J. Immunol. 191, 527–532. 10.4049/jimmunol.1301035 23825387

[B16] Jastreboff A. M. Kotz C. M. Kahan S. Kelly A. S. Heymsfield S. B. (2019). Obesity as a disease: the obesity society 2018 position statement. Obesity 27, 7–9. 10.1002/oby.22378 30569641

[B17] Jia L. Vianna C. R. Fukuda M. Berglund E. D. Liu C. Tao C. (2014). Hepatocyte toll-like receptor 4 regulates obesity-induced inflammation and insulin resistance. Nat. Commun. 5, 3878 10.1038/ncomms4878 24815961 PMC4080408

[B18] Kim J. K. Kim Y. J. Fillmore J. J. Chen Y. Moore I. Lee J. (2001). Prevention of fat-induced insulin resistance by salicylate. J. Clin. Invest. 108, 437–446. 10.1172/JCI11559 11489937 PMC209353

[B19] Lackey D. E. Olefsky J. M. (2016). Regulation of metabolism by the innate immune system. Nat. Rev. Endocrinol. 12, 15–28. 10.1038/nrendo.2015.189 26553134

[B20] Lee Y. H. Petkova A. P. Granneman J. G. (2013). Identification of an adipogenic niche for adipose tissue remodeling and restoration. Cell Metabol. 18, 355–367. 10.1016/j.cmet.2013.08.003 PMC4185305 24011071

[B21] Lumeng C. N. Bodzin J. L. Saltiel A. R. (2007). Obesity induces a phenotypic switch in adipose tissue macrophage polarization. J. Clin. Invest. 117, 175–184. 10.1172/JCI29881 17200717 PMC1716210

[B22] Mantovani A. Sica A. Sozzani S. Allavena P. Vecchi A. Locati M. (2004). The chemokine system in diverse forms of macrophage activation and polarization. Trends Immunol. 25, 677–686. 10.1016/j.it.2004.09.015 15530839

[B23] Moller D. E. Kaufman K. D. (2005). Metabolic syndrome: a clinical and molecular perspective. Annu. Rev. Med. 56, 45–62. 10.1146/annurev.med.56.082103.104751 15660501

[B24] Narender T. Puri A. Shweta T. Saxena R. Bhatia G. (2006). 4-hydroxyisoleucine an unusual amino acid as antidyslipidemic and antihyperglycemic agent. Bioorg. Med. Chem. Lett. 16, 293–296. 10.1016/j.bmcl.2005.10.003 16246556

[B25] Nicolas L. Olivier M. C. Yves H. Nico V. R. Cani P. D. Leclercq I. A. (2010). Kupffer cell activation is a causal factor for hepatic insulin resistance. Am. J. Physiol. Gastrointest. Liver Physiol. 298, G107–G116. 10.1152/ajpgi.00391.2009 19875703

[B26] Olefsky J. M. Glass C. K. (2010). Macrophages, inflammation, and insulin resistance. Annu. Rev. Physiol. 72, 219–246. 10.1146/annurev-physiol-021909-135846 20148674

[B27] Pal D. Dasgupta S. Kundu R. Maitra S. Das G. Mukhopadhyay S. (2012). Fetuin-A acts as an endogenous ligand of TLR4 to promote lipid-induced insulin resistance. Nat. Med. 18, 1279–1285. 10.1038/nm.2851 22842477

[B28] Patsouris D. Li P. P. Thapar D. Chapman J. Olefsky J. M. Neels J. G. (2008). Ablation of CD11c-positive cells normalizes insulin sensitivity in obese insulin resistant animals. Cell Metabol. 8, 301–309. 10.1016/j.cmet.2008.08.015 PMC2630775 18840360

[B29] Pillon N. J. Bilan P. J. Fink L. N. Klip A. (2013). Cross-talk between skeletal muscle and immune cells: muscle-derived mediators and metabolic implications. Am. J. Physiol. Endocrinol. Metab. 304, E453–E465. 10.1152/ajpendo.00553.2012 23277185

[B30] Pingping L. Min L. Nguyen M. T. A. Eun Ju B. Justin C. Daorong F. (2010). Functional heterogeneity of CD11c-positive adipose tissue macrophages in diet-induced obese mice. J. Biol. Chem. 285, 15333–15345. 10.1074/jbc.M110.10026 20308074 PMC2865288

[B31] Rawat A. K. Korthikunta V. Gautam S. Pal S. Tadigoppula N. Tamrakar A. K. (2014). 4-Hydroxyisoleucine improves insulin resistance by promoting mitochondrial biogenesis and act through AMPK and Akt dependent pathway. Fitoterapia. 99, 307–317. 10.1016/j.fitote.2014.10.006 25454462

[B32] Saltiel A. R. Olefsky J. M. (2017). Inflammatory mechanisms linking obesity and metabolic disease. J. Clin. Invest. 127, 1–4. 10.1172/JCI92035 28045402 PMC5199709

[B33] Sauvaire Y. Girardon P. Baccou J. C. Ristérucci A. M. (1984). Changes in growth, proteins and free amino acids of developing seed and pod of fenugreek. Phytochemistry 23, 479–486. 10.1016/S0031-9422(00)80363-5

[B34] Schneemann M. Schoedon G. (2002). Species differences in macrophage NO production are important. Nat. Immunol. 3, 102 10.1038/ni0202-102a 11812978

[B35] Sergent D. Wang Q. Sasaki N. A. Ouazzani J. (2008). Synthesis of hydantoin analogues of (2S,3R,4S)-4-hydroxyisoleucine with insulinotropic properties. Bioorg. Med. Chem. Lett. 18, 4332–4335. 10.1016/j.bmcl.2008.06.081 18621529

[B36] Sharma R. D. (1986). Effect of fenugreek seeds and leaves on blood glucose and serum insulin responses in human subjects. Nutr. Res. 6, 1353–1364. 10.1016/S0271-5317(86)80020-3

[B37] Shi H. Kokoeva M. V. Inouye K. Tzameli I. Yin H. Flier J. S. (2006). TLR4 links innate immunity and fatty acid-induced insulin resistance. J. Clin. Invest. 116, 3015–3025. 10.1172/JCI28898 17053832 PMC1616196

[B38] Singh A. B. Tamarkar A. K. Narender T. Srivastava A. K. (2010). Antihyperglycaemic effect of an unusual amino acid (4-hydroxyisoleucine) in C57BL/KsJ-db/db mice. Nat. Prod. Res. 24, 258–265. 10.1080/14786410902836693 20140804

[B39] Stone R. (2008). Biochemistry. Lifting the veil on traditional Chinese medicine. Science 319, 709–710. 10.1126/science.319.5864.709 18258866

[B40] Tanaka M. Ikeda K. Suganami T. Komiya C. Ochi K. Shirakawa I. (2014). Macrophage-inducible C-type lectin underlies obesity-induced adipose tissue fibrosis. Nat. Commun. 5, 4982 10.1038/ncomms5982 25236782

[B41] Wada J. Makino H. (2016). Innate immunity in diabetes and diabetic nephropathy. Nat. Rev. Nephrol. 12, 13–26. 10.1038/nrneph.2015.175 26568190

[B42] Weisberg S. P. Mccann D. Desai M. Rosenbaum M. Leibel R. L. Ferrante A. W. Jr. (2003). Obesity is associated with macrophage accumulation in adipose tissue. J. Clin. Invest. 112, 1796–1808. 10.1172/JCI19246 14679176 PMC296995

[B43] Xu H. Barnes G. T. Yang Q. Tan G. Yang D. Chou C. J. (2003). Chronic inflammation in fat plays a crucial role in the development of obesity-related insulin resistance. J. Clin. Invest. 112, 1821–1830. 10.1172/JCI19451 14679177 PMC296998

[B44] Ying W. Fu W. Lee Y. S. Olefsky J. M. (2020). The role oThe role of macrophages in obesity-associated islet inflammation and β-cell abnormalities. Nat. Rev. Endocrinol. 16, 81–90. 10.1038/s41574-019-0286-3 31836875 PMC8315273

[B45] Yuan M. Konstantopoulos N. Lee J. Hansen L. Li Z. W. Karin M. (2001). Reversal of obesity- and diet-induced insulin resistance with salicylates or targeted disruption of Ikkbeta. Science 293, 1673–1677. 10.1126/science.1061620 11533494

[B46] Zhang X. Laubach V. E. Alley E. W. Edwards K. A. Sherman P. A. Russell S. W. (1996). Transcriptional basis for hyporesponsiveness of the human inducible nitric oxide synthase gene to lipopolysaccharide/interferon-gamma. J. Leukoc. Biol. 59, 575–585. 10.1002/jlb.59.4.575 8613707

[B47] Zhang F. Zarkada G. Han J. Li J. Dubrac A. Ola R. (2018). Lacteal junction zippering protects against diet-induced obesity. Science 361, 599–603. 10.1126/science.aap9331 30093598 PMC6317738

