# Supplementary material for: 4-Hydroxyisoleucine Alleviates Macrophage-Related Chronic Inflammation and Metabolic Syndrome in Mice Fed a High-Fat Diet
Source: Front Pharmacol. 2021 Jan 21;11:606514. doi: 10.3389/fphar.2020.606514 (PMC7858251; doi:10.3389/fphar.2020.606514)
Supplement: Supplementary file 1 [file datasheet1.docx]

Supplementary Material

**4-Hydroxyisoleucine alleviates macrophage-related chronic inflammation and metabolic syndrome in mice fed a high-fat diet**


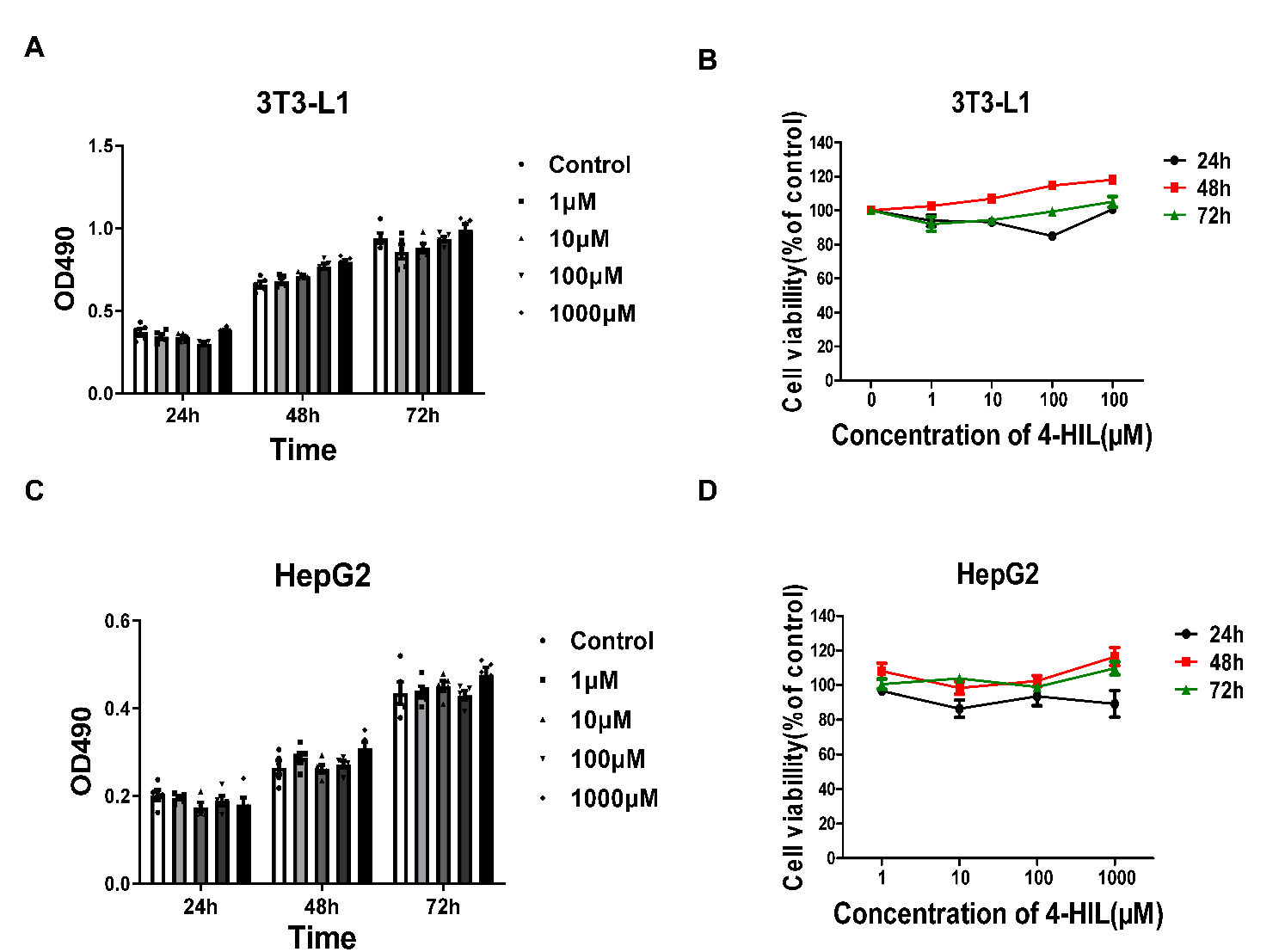


**Supplementary Figure 1.** Cells were seeded into 96-well culture plates at a density 4000 cells/well. After 24, 48 and 72 hours, cell viability was measured using MTT reagent and DMSO. MTT reduction was quantified by measuring the absorbance at 490 nm (OD = optical density).


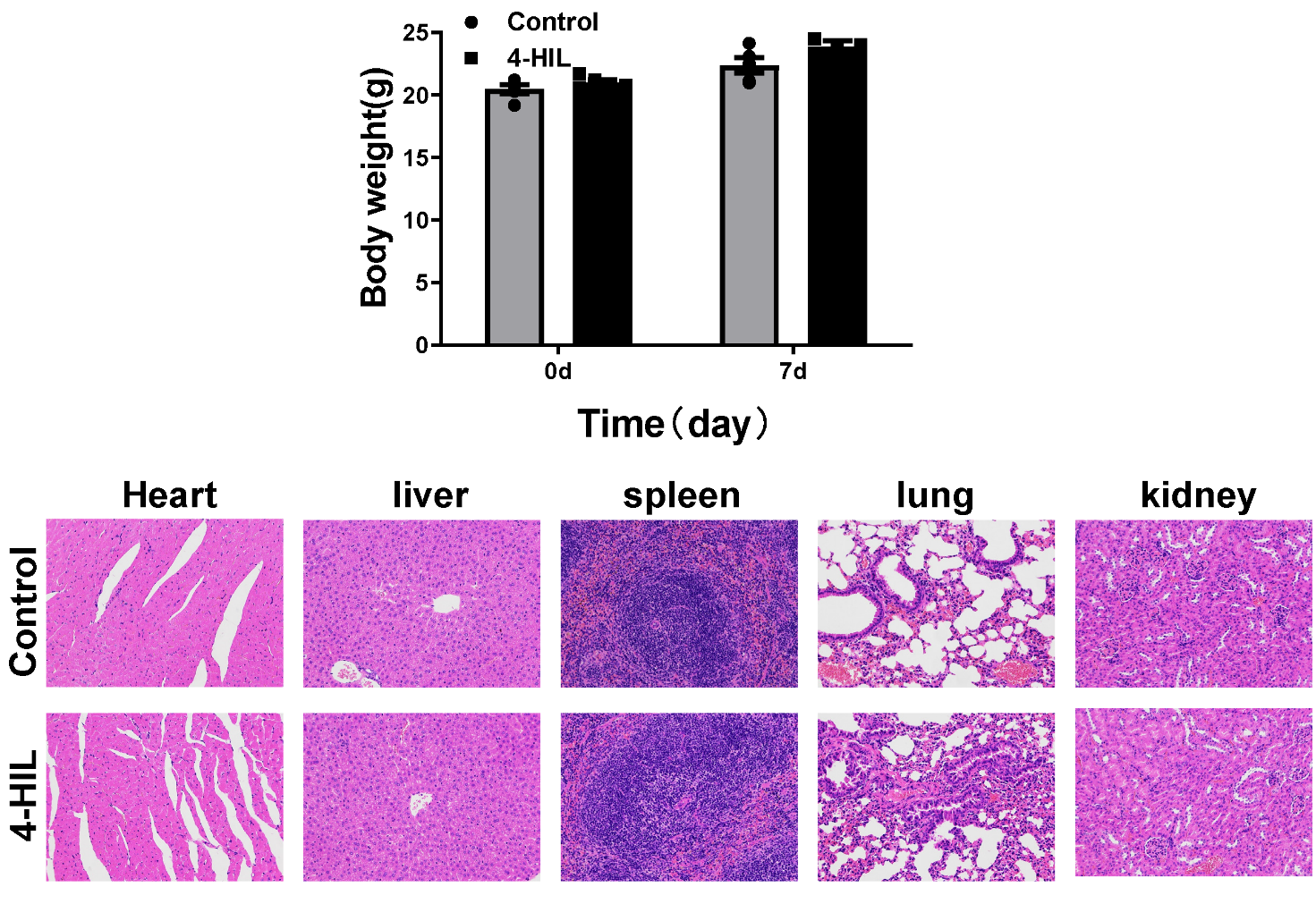


**B**

**A**

**Supplementary Figure 2.** (A) biocompatibility of the 4-HIL with reference to mice weight; (B) histopathological images by H&E staining to analyze systematic toxicity (heart, liver, spleen, lung, and kidney) of mice with 7 days posttreatment.


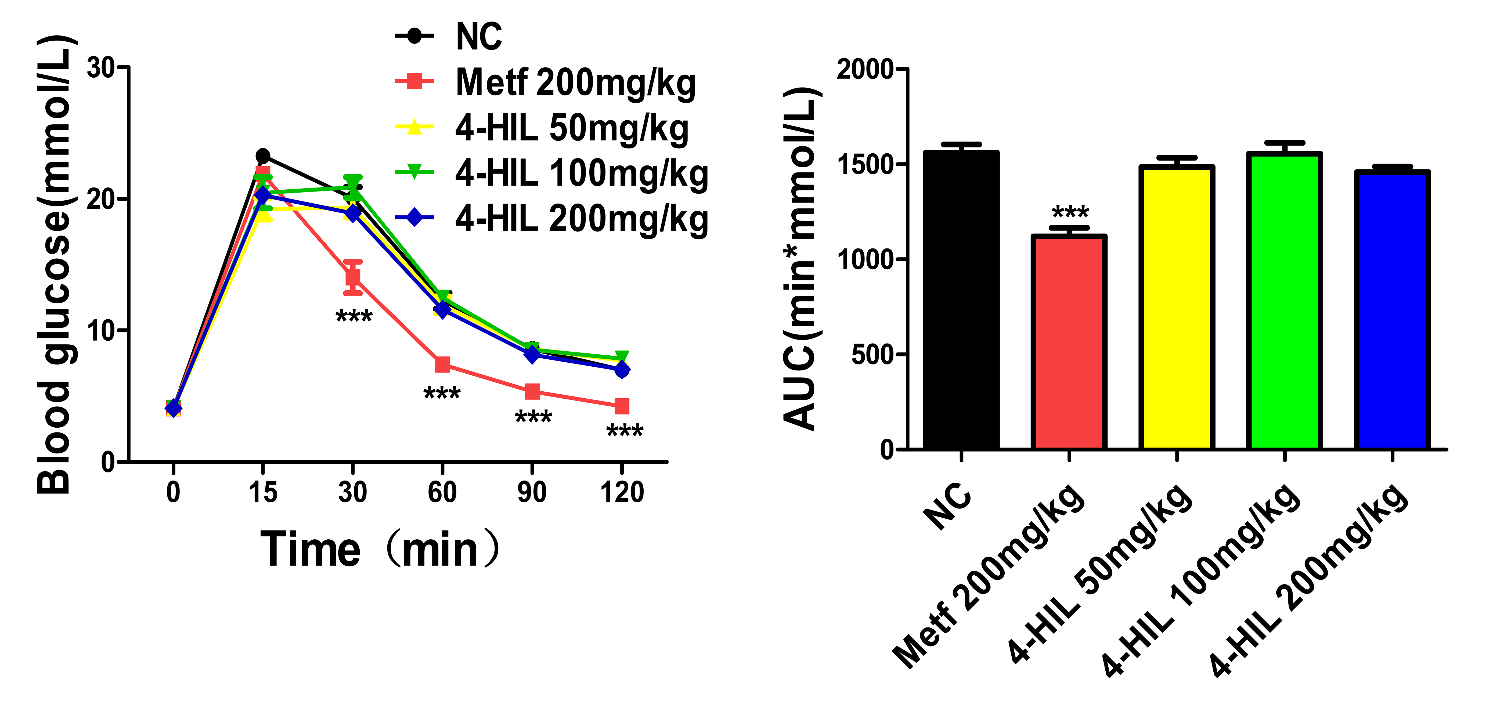


**Supplementary Figure 3.** This experiment assessed the effect of the administration of 4-HIL on glucose tolerance in normal C57BL/6 mice, with the intent of evaluating the hypoglycemic effect of the drug. Glucose solution (2 g/kg) was administered simultaneously with 4-HIL and blood glucose levels were determined 15, 30, 60, 90, and 120 min after administration. The area under the blood glucose time curve (AUC) was calculated. * difference compared with the normal control (NC) group.


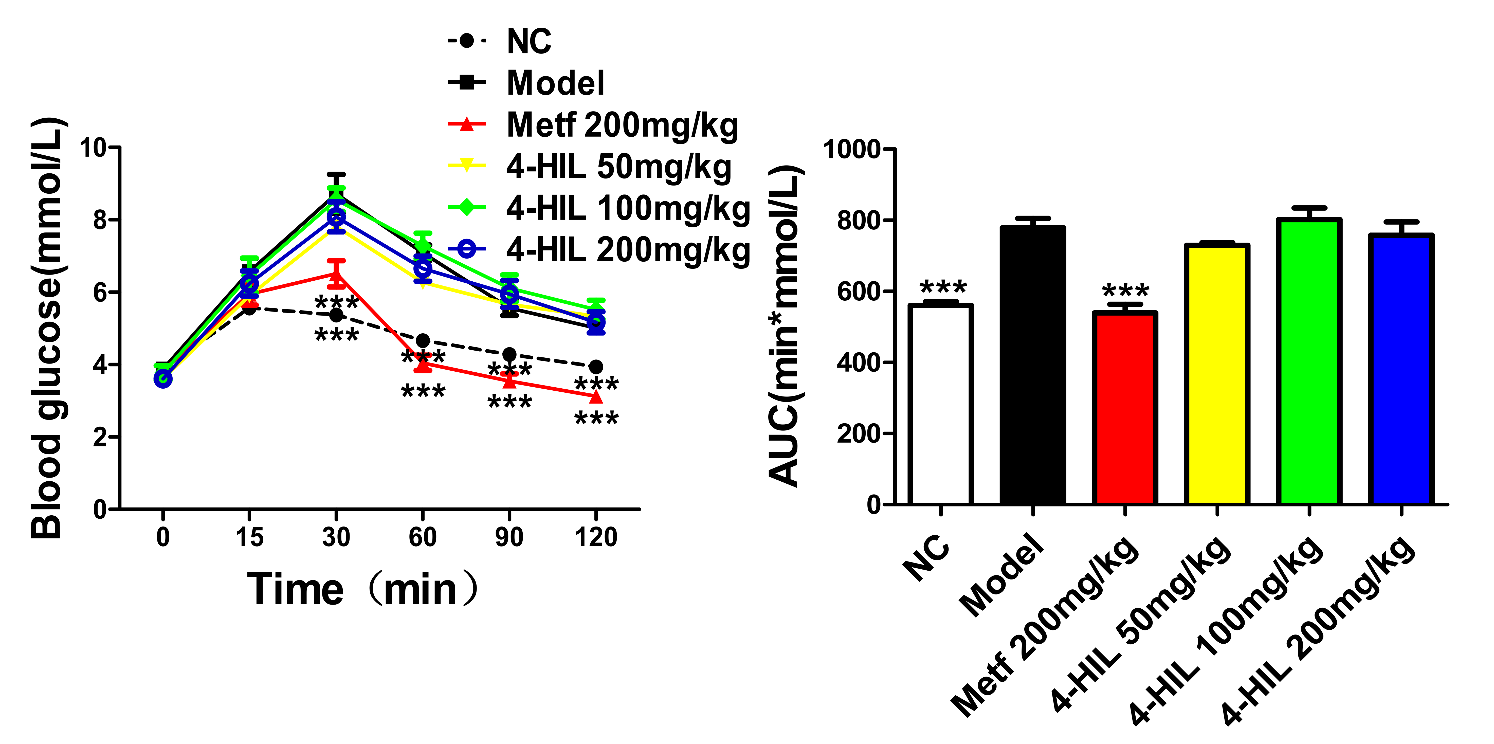


**Supplementary Figure 4.** The effect of 4-HIL on blood glucose in epinephrine-induced acute hyperglycemic C57BL/6 mice was measured for 5 consecutive days. 4-HIL was administered for the 5 consecutive days. After the last 10-min administration, except for the normal control (NC) group, the other groups were intraperitoneally injected with epinephrine (200 μg/kg), and blood glucose levels were measured 15, 30, 60, 90, and 120 min after administration were measured. The area under the blood glucose time curve (AUC) was calculated. * difference compared to the model group.


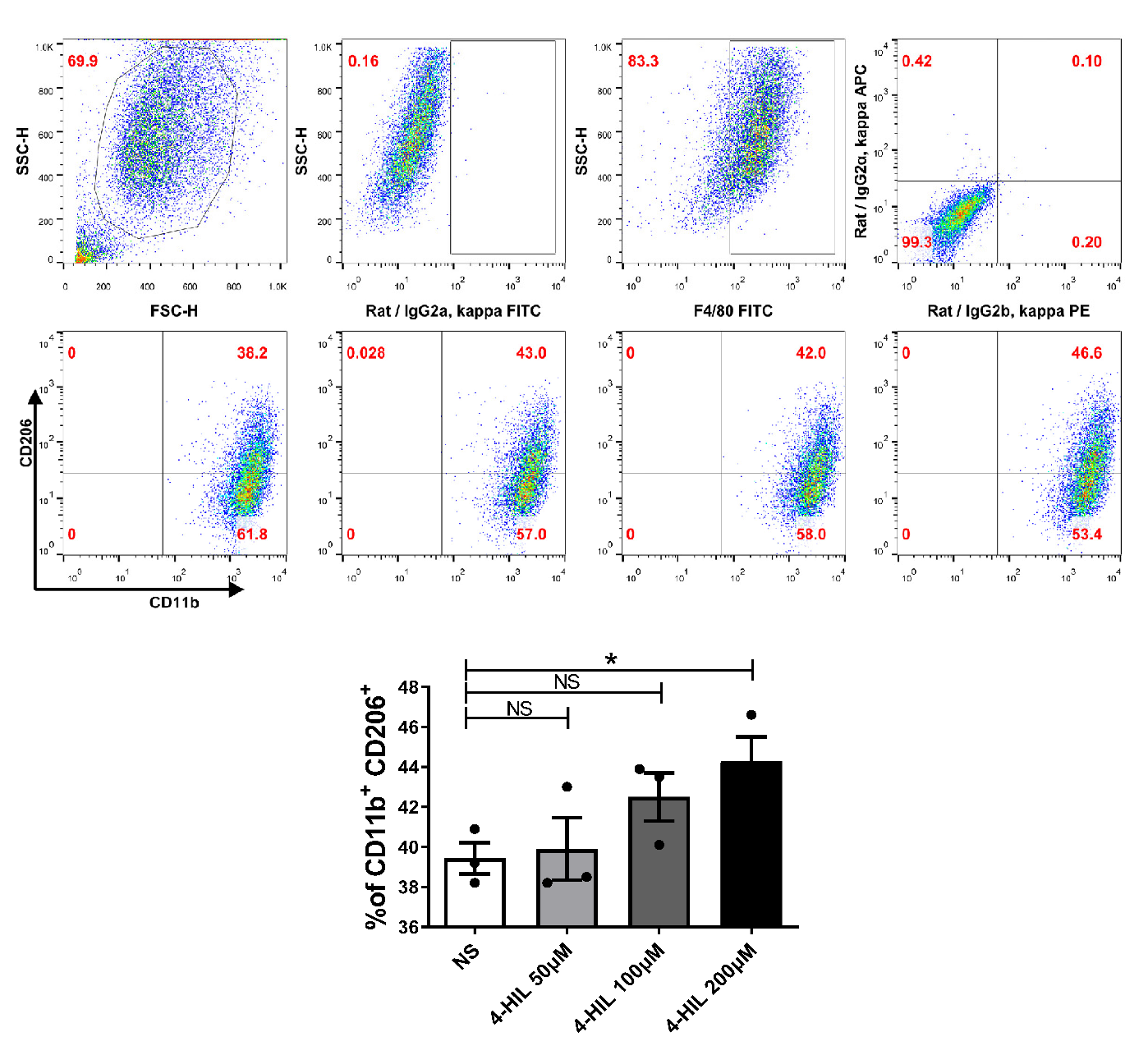


**Supplementary Figure 5** .To generate M2 macrophages, bone marrow cells were cultured in the presence of M-CSF (20 ng/mL) for 7 days, at the 6 days and 7 days IL-13 (20 ng/mL), IL4 (20 ng/mL) were add. The cells were inoculated into wells of a 6-well plate at the density of 2 × 10^5^ / well in a 6-well plate. The cells were incubated overnight (12 h) in serum-free DMEM. After this period of synchronization, 4-HIL was added to the culture for 24 h. Control cells were not treated. Both groups of macrophages were examined by flow cytometry.

**Supplementary Table 1. Primers used in this study**

| Name | Sequence |
| --- | --- |
| 36B4 Forward | 5’-AGTGCTCGACATCACAGAGCA-3’ |
| 36B4 Reverse | 5’-GCGCTTGTACCCATTGATGA-3’ |
| TNF-α Forward | 5’-AGGGTCTGGGCCATAGAACT-3’ |
| TNF-α Reverse | 5’-CCACCACGCTCTTCTG TCTA-3’ |
| IL-6 Forward | 5’-CTCTGCAAGAGACTTCCATCCAGT-3’ |
| IL-6 Reverse | 5’-GAAGTAGGG AAGGCCGTGG-3’ |
| IL-1β Forward | 5’-TTGAAGAAGAGCCCATCCTC-3’ |
| IL-1β Reverse | 5’-CAGCTCATATGGGTCCGAC-3’ |
| NF-κ B Forward | 5’-TGTCAGAGCCCTTGAAACTG-3’ |
| NF-κ B Reverse | 5’-CTGTGGGTAGGATTTCTTGT-3’ |
| MCP-1 Forward | 5’-TCACTGAAGCCAGCTCTCTCT-3’ |
| MCP-1 Reverse | 5’-GTGGGGCGTTAACTGCAT-3’ |
| PAI-1 Forward | 5’-TCAGCCCTTGCTTGCCTCAT-3’ |
| PAI-1 Reverse | 5’-GCATAGCCAGCACCGAGGA-3’ |
